# Effects of communication style, anthropomorphic setting and individual differences on older adults using voice assistants in a health context

**DOI:** 10.1186/s12877-022-03428-2

**Published:** 2022-09-15

**Authors:** Runting Zhong, Mengyao Ma

**Affiliations:** 1 School of Business, Jiangnan University, Wuxi, Jiangsu 214122 People’s Republic of China; 2 School of Management, University of Science and Technology of China, Hefei, Anhui 230026 People’s Republic of China

**Keywords:** Older adults, Internet of things (IoT), Technology acceptance, Trust, Voice assistant

## Abstract

**Background:**

Voice assistants enable older adults to communicate regarding their health as well as facilitate ageing in place. This study investigated the effects of communication style, anthropomorphic setting, and individual differences on the trust, acceptance, and mental workload of older adults using a voice assistant when communicating health issues.

**Methods:**

This is a mixed-methods study utilising both quantitative and qualitative methods. One hundred and six older adults (M = 71.8 years, SD = 4.6 years) participated in a 2 (communication style: social- vs. task-oriented; between-subject)$\times$ 2 (anthropomorphic setting: ordinary profession vs. medical background; within-subject) mixed design experiment. The study used multivariate analysis of variance (MANOVA) to examine the effects of communication style, anthropomorphic setting of the voice assistant, and participants’ use frequency of digital devices on the trust, technology acceptance, and mental workload of older adults using a voice assistant in a health context. End-of-study interviews regarding voice assistant use were conducted with participants. Qualitative content analyses were used to assess the interview findings about the communication content, the more trustworthy anthropomorphic setting, and suggestions for the voice assistant.

**Results:**

Communication style, anthropomorphic setting, and individual differences all had statistically significant effects on older adults’ evaluations of the voice assistant. Compared with a task-oriented voice assistant, older adults preferred a social-oriented voice assistant in terms of trust in ability, integrity, and technology acceptance. Older adults also had better evaluations for a voice assistant with a medical background in terms of trust in ability, integrity, technology acceptance, and mental workload. In addition, older adults with more experience using digital products provided more positive evaluations in terms of trust in ability, integrity, and technology acceptance.

**Conclusions:**

This study suggests that when designing a voice assistant for older adults in the health context, using a social-oriented communication style and providing an anthropomorphic setting in which the voice assistant has a medical background are effective ways to improve the trust and acceptance of older adults of voice assistants in an internet-of-things environment.

**Supplementary Information:**

The online version contains supplementary material available at 10.1186/s12877-022-03428-2.

## Introduction

Off-the-shelf voice assistants (for example, Amazon Alexa and Google Home) provide a cost-effective means for older adults to communicate health information at home and thus facilitate ageing in place. In the internet-of-things (IoT) era, IoT voice assistants have become a gateway for users to connect with smart spaces [1]. People can converse with their IoT smart devices, such as smart lights or smart TVs, as with a friend via an IoT voice assistant in a smart home environment [1]. An IoT voice assistant could act as a health assistant for older adults, such as by providing updated dietary health information [2], asking health-related questions [3], reducing loneliness [4], and encouraging fitness exercise [5, 6], all of which could reduce pressure on the health care system during the coronavirus disease-2019 (COVID-19) pandemic [7]. In addition, an IoT voice assistant benefits older adults because interaction with voice assistants is more natural for such adults than interaction with smartphones or computers [2].

The challenges in designing a voice assistant for older adults involve the problems of having trust in the voice assistant, technology acceptance and mental workload. Older adults may abandon using the voice assistant as they may not consider it trustworthy, specifically in sensitive contexts (for example, health care) [8]. According to Mayer et al., the three factors that determine trustworthiness are the ability, integrity, and benevolence of the trustee [9]. “Ability” is defined as a “group of skills, competencies, and characteristics that enable a party to have influence within some specific domain”. “Integrity” means “the trustee adheres to a set of principles that the trustor finds acceptable”. “Benevolence” is “the extent to which a trustee is believed to want to do good to the trustor, aside from an egocentric profit motive” [9]. Technology acceptance is also important for older adults because the current generation of older adults who did not grow up using this type of technology have more difficulty understanding technological functions and instructions than younger people [10]. Mental workload is also of interest since it can reflect older adults’ frustration level in interacting with a voice assistant through subjective self-assessment [11]. “Mental workload” is defined as the degree to which tasks require limited physical and psychological resources of human beings [12]. Trust, technology acceptance, and mental workload are frequently used as indicators of user experience in human–computer studies, while higher trust, technology and lower mental workload indicate better performance with respect to user experience [13–15].

The objective of this study is to understand older adults’ requirements and preferences regarding voice assistants in a health-related context. The study examines the voice assistant as a tool to provide health information and guide older adults in following a healthy lifestyle. We designed an experiment to investigate the effects of anthropomorphic setting (professional background vs. ordinary occupation), voice assistant communication style (social- vs. task-oriented), and individual differences between older adults in their trust, acceptance, and mental workload while interacting with a health-related IoT voice assistant.

### Related research and hypothesis

#### Effect of communication style

The communication style of a voice assistant may affect user evaluation. People are more likely to trust artificial agents with a happy tone and more familiar language styles [16, 17]. Studies have examined the effect of voice assistant communication style in various scenarios. Bickmore found that engaging in small talk with a voice assistant can be effective in settings such as customer service, health care, and weather information, but it may not contribute much in more serious settings, such as financial transactions or military training [18]. In an online shopping task, a social-oriented voice assistant could enhance trust at a website for older adults with high internet competency, but for those with low internet competency, a task-oriented voice assistant provided more significant cooperative and functional advantages [14].

In actual physician–patient communication, physicians seem to use a more task-oriented than social-oriented communication style. Yoo et al. found that text messaging between adolescents with asthma and clinicians was focused more on task-focused behaviours (345 times in 314 text messages) over socioemotional behaviours (237 times in 314 text messages) [19]. Chinese medical personnel seldom use affective descriptors but, rather, focus on technical tasks [20]. Bradley et al. found that the consultative style (i.e., doctor and patient as a collaborative team) brings higher satisfaction to patients than the authoritative style (i.e., doctor as an expert; more task-oriented) [21]. If a voice assistant is to behave as a health agent for older adults, it could be designed to communicate similarly to a real doctor. In this study, our focus is on examining whether the communication style (social-oriented vs. task-oriented) of voice assistants affects older adults’ evaluation of voice assistants in the health care context. Thus, we propose the following hypothesis:

H1: The communication style of a voice assistant (social- vs. task-oriented) has a significant effect on the trust, acceptance, and mental workload of older adults in the health-related context.

### Effect of anthropomorphic setting

Previous studies have examined the effect of anthropomorphic setting on trust in robots. People consider voice assistants with female voices to be friendlier [22] and are more inclined to use voice assistants with a familiar appearance and mannerisms similar to those of humans [23]. Mo et al. found that older adults perceived social robots with in-family attributes (i.e., where the robot is viewed as a member of the older adult’s family) as more social and trustworthy than those without family attributes (i.e., where the robot is not a member of the older adult’s family) [24]. Zhao and Rau found that young people preferred a voice assistant that combined both work and life functions rather than one that handled either item separately [13]. Biermann et al. found that robots with a technical appearance with only the necessary mechanical structures were trusted more than anthropomorphic robots or robots in the care context [25].

In the field of health, people may place special trust in “experts”. A study found that consumers chose expert labels as the most- or second-most trustworthy among food product labels [26]. In China, doctors are regarded as experts and rarely challenged by patients [27]. In this study, we use real-life experience as a metaphor to design voice assistants as doctors, and it can be assumed that the “professional background” of the voice assistant may affect the user experience evaluation of the voice assistant. Thus, we propose the following hypothesis:

H2: The anthropomorphic setting of a voice assistant (professional background vs. ordinary occupation) has a significant effect on the trust, acceptance, and mental workload of older adults in the health-related context.

### Effect of individual differences

Older adults’ attitudes and behaviours towards voice assistants may also be affected by individual differences, such as age, income, living situation and prior experience with technology. Attitudes towards voice assistants may change as adults grow older and their needs change [28]. Low-income older adults who use voice assistants like the ability to by notified regarding harmful events, such as changes in health indicators [29]. The lonelier that an older adult is, the more likely he or she is to perceive a voice assistant as human, and as the older adults increasingly anthropormorphise the voice assistant, their loneliness decreases [4]. Ghorayeb et al. found that among older adults who have already tried smart home monitoring technologies, acceptance increases over time and with use [30]. Such adults expressed fewer concerns than inexperienced smart home participants regarding privacy, trust, and usability and more concerns regarding utility [30]. Therefore, investigating the effect of participants’ individual differences (e.g., age, gender, living situation, prior technology use) on the user experience of health-related voice assistants is of interest to us. Thus, we propose the following:

H3: Individual differences among older adults (for example, age, gender, living situation, and prior technology use) have a significant effect on trust, acceptance, and mental workload in the health-related context.

## Materials and methods

### Experimental design

A 2 (communication style: social- vs. task-oriented; between-subject)$\times$ 2 (anthropomorphic setting: ordinary profession vs. medical background; within-subject) mixed design was adopted. Demographic variables (i.e., age, gender, living situation, use experience of voice assistant, and frequency of using digital products) of the older adults were set as covariates. The sample size required to achieve a statistical power of 0.80 should not be less than 98 (effect size f = 0.25, $\alpha$ = 0.05, 1-$\beta$ = 0.8, number of groups = 2, number of measurements = 2, Corr among rep measures = 0.5), as calculated using G*Power 3.1 [31].

**Fig. 1:**
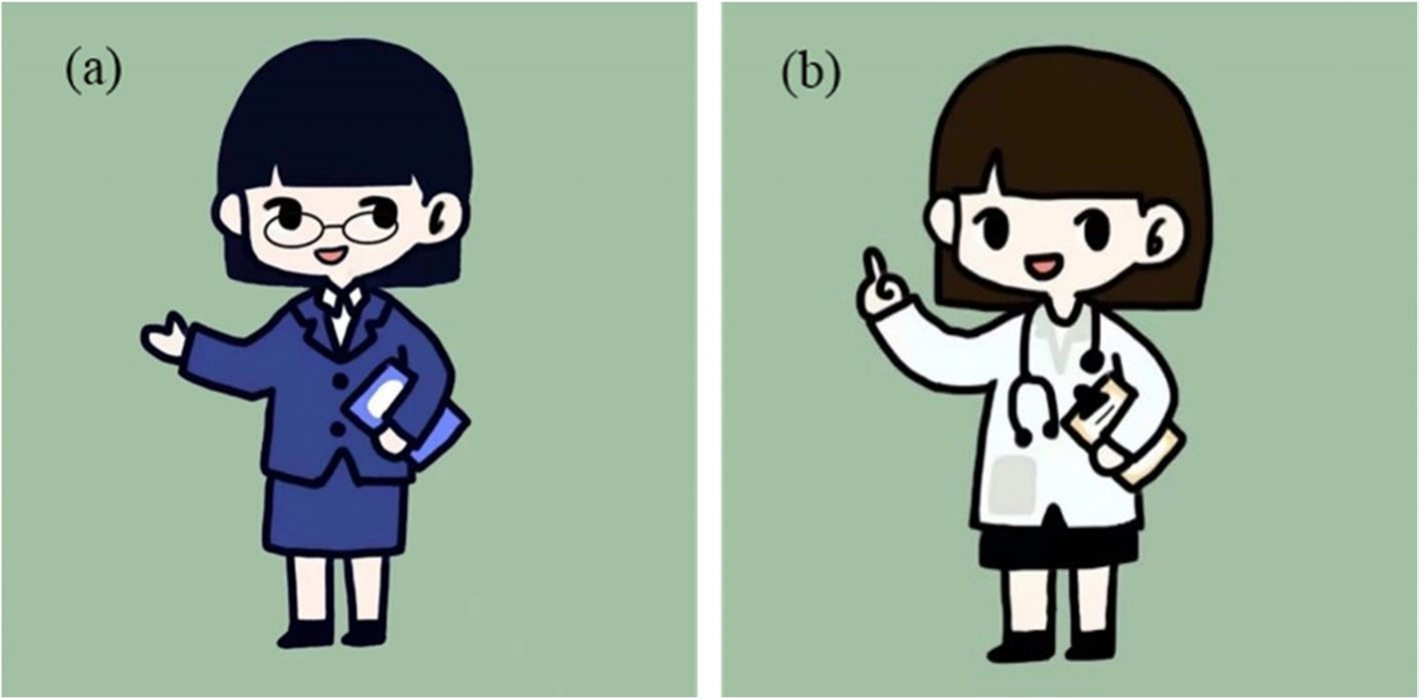
Anthropomorphic settings for voice assistants (**a**) Xiao Li and (**b**) Doctor Li

Regarding anthropomorphic setting, the voice assistant wore an introduction card (introducing the voice assistant’s work address, title and occupation). This study used cartoon characters for the voice assistants so that the participants could sense their occupational differences directly through the different uniforms. By reviewing the relevant literature [22, 32], we found that users think that female voices are amiable and gentle. To control for the gender variables of the voice assistant in the experiment, female characters were selected. Chinese voice assistant devices generally use Xiao + words as the wakeup words. For example, the wakeup word for the voice assistant produced by Baidu is XiaoDu, while that for the voice assistant produced by Xiaomi is Xiaoai. In addition, in terms of the "name" itself, the "Xiao + word" name is the most popular among users [33]. Because Li is a common Chinese surname, the wakeup word for the voice assistant was set as Xiao Li in the experiment. We used the name Xiao Li for the voice assistant with an ordinary profession and the name Doctor Li for the voice assistant with a medical background. The anthropomorphic settings of the voice assistants are presented in Fig. 1.

The task-oriented communication style of the voice assistant was formal. Its interaction with the user was limited to providing instructions and other information to help the user complete a task. There was no social conversation beyond the initial greeting of the initial wakeup call. In contrast, the social-oriented communication style of the voice assistant was conversational. In addition to providing necessary guidance and information, the voice assistants maintained informal conversations through small talk, questions, exclamatory feedback, and encouragement [4]. We obtained common questions regarding relevant scenes from older adults in the community and then searched the web for relevant questions to obtain answers. First, we extracted task-oriented answers according to the requirements and then transformed the task-oriented answers into social-oriented ones by adding small talk, caring statements, and mood words. Seven participants (who did not participate in the formal experiment) participated in the pre-experiment, which verified that the experiment could be completed smoothly and the script content and experiment process correctly understood. An example of a conversation is presented in Table 1.

**Table 1:** Design of the voice assistant interaction (Task Script Example: Diet)

Task-oriented	Social-oriented
**VA:** Hello, this is Xiao Li (Doctor Li). Can I help you? **P:** Are lamb and chestnuts exclusive? **VA:** Lamb and chestnuts are mutually exclusive **P:** Can fish and tofu be eaten together? **VA:** Fish and tofu can be eaten together **P:** Is eating tomatoes good for diabetes? **VA:** It is good. If you need more relevant information, you can ask me	**VA:** Hi! This is Xiao Li (Doctor Li), I am so happy to see you today! Do you have any questions for me? I look forward to helping you answer them! **P:** Are lamb and chestnuts exclusive? **VA:** Lamb and chestnuts are mutually exclusive, so do not eat them together! **P:** Can fish and tofu be eaten together? **VA:** Fish and tofu can be eaten together. They are delicious together. Would you like to try them? **P:** Is eating tomatoes good for diabetes? **VA:** Good; tomatoes have high nutritional value. I also know many other foods that are good for diabetes. If you want to know, I will tell you!

*VA*, voice assistant, *P*, participant

### Apparatus

We converted the script to Portable Document Format (PDF) and then implemented the reading function using the native language package of Microsoft Edge (Microsoft Xiaoxiao Online (Natural)). The recording sounds like the voice of a young woman. Subsequently, we used recording software to record all the scripts.

The “Wizard of Oz” method was applied. The participants interacted with a computer that they believed to be autonomous but that was actually operated by a person [34]. This method has been extensively used in human–computer interaction studies [13, 35]. The “Wizard of Oz” method was used instead of interacting with an off-the-shelf voice assistant for the following reasons. First, the accuracy of the off-the-shelf voice assistant is insufficient, and there may be “search bubbles” during the interaction [36]. Second, we set the communication style as a between-subject variable and designed the dialogue scripts accordingly. Thus, it was more convenient to control the voice assistant using “Wizard of Oz”.

**Fig. 2:**
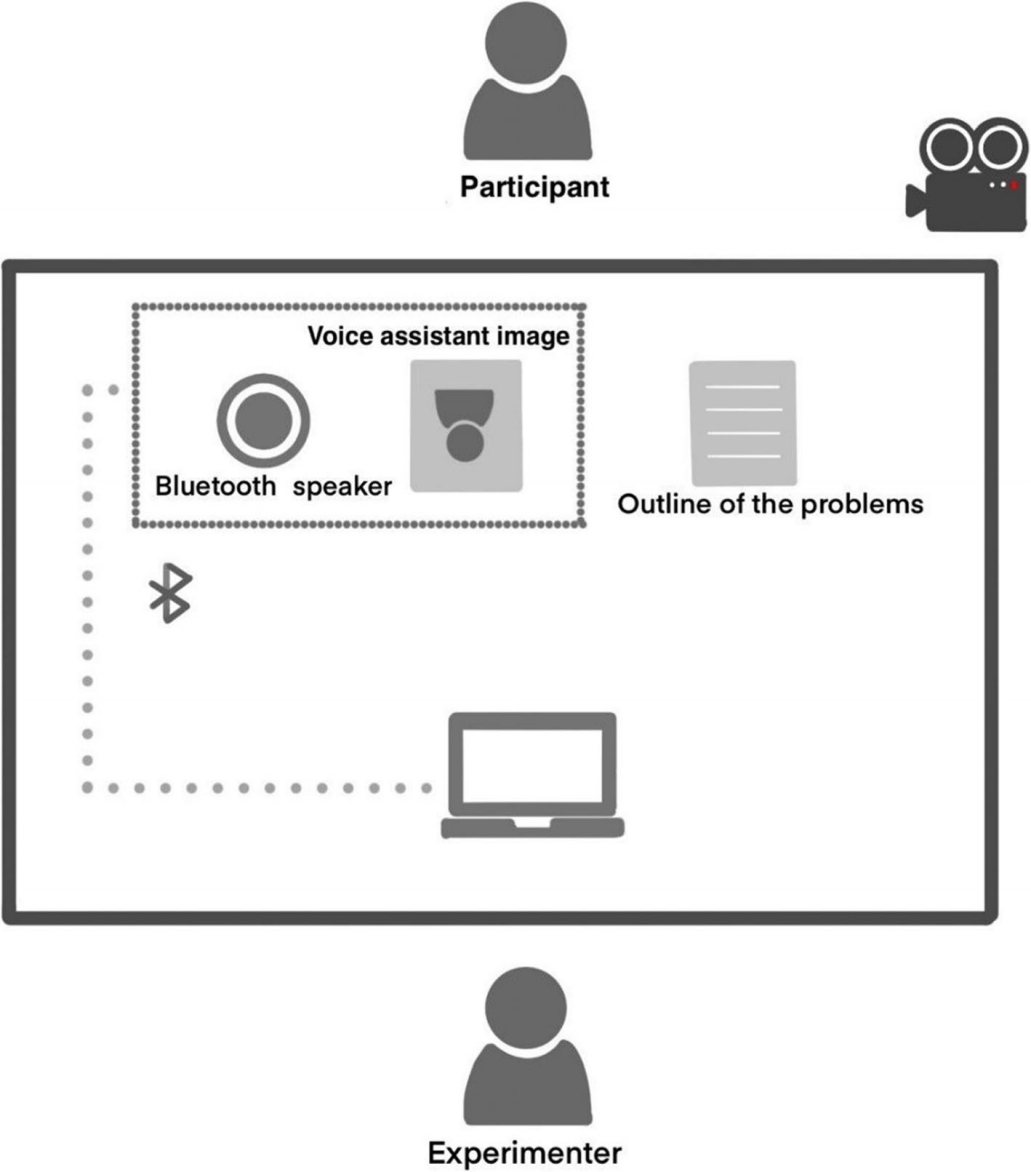
Experimental setup

During the experiment, we stored the recording on a computer and played the voice assistant’s voice through a Huawei mini–Bluetooth speaker (Model CM510, Huawei Technologies Co., China) when interacting with older adults. The experimental setup is shown in Fig. 2.

### Participants

One hundred and six older adults (61–84 years old) were recruited between January and February 2021 through word of mouth from communities in Panzhihua City (urban), Sichuan Province, China. In 2021, the city's domestic product (GDP) was 113.395 billion yuan (1 yuan = 0.147 US dollars), which ranked 65th among China's 333 mainland prefecture-level cities [37]. The per capita GDP of Panzhihua City is 93,500 yuan, while the per capita GDP of China is 80,976 yuan [38]. The inclusion criteria were age ≥ 60 years, living independently in the community, able to communicate in Mandarin Chinese or Sichuanese and not having visual and hearing impairments. There was no professional psychological screening. However, at the beginning of the experiment, the experimenter communicated with the participants to confirm that the participants had relatively healthy and normal psychological cognitive function. As noted, an exclusion criterion was the inability to communicate verbally in Mandarin Chinese or Sichuanese. We recruited a heterogeneous group of older adults of different ages, living situations, and technology use experience. The study’s ethics were approved by the university of the authors.

### Tasks and procedure

The experimental procedure lasted 25 min for each participant. First, the participants signed consent forms and completed a questionnaire eliciting demographic information, including age, gender, living situation, voice assistant use experience, and weekly use of digital products. Next, the experimenter showed the participant how to use the voice assistant. Subsequently, each participant performed three tasks that required interacting with the voice assistant. Each task lasted approximately two min, and all participants were required to read the script shown in Table 1 (Diet), Appendix Table 1 (Exercise) and A2 (Medical consultation). The participants asked the voice assistant the same set of questions. The three tasks were as follows:

#### Task 1: Diet

The older adults were instructed to ask the voice assistant questions related to food, including food combinations, therapeutic effect, and the context in which food is consumed. Finally, the older adults provided corresponding responses based on the answers provided by the voice assistant.

#### Task 2: Exercise

The older adults asked the voice assistant questions related to their fitness. Subsequently, they asked the voice assistant how to perform a chest expansion exercise. Thereafter, they followed the instructions of the voice assistant and performed the chest expansion exercise according to the provided rhythm.

#### Task 3: Medical consultation

The older adults asked the voice assistant questions regarding cold symptoms, suggestions for medical treatment after the illness, and preventive care. Finally, the older adults furnished corresponding responses based on the answers provided by the voice assistant.

**Fig. 3:**
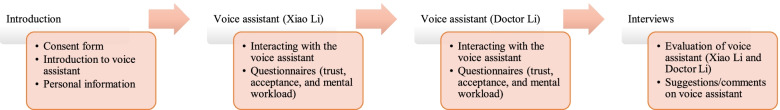
Experimental procedure

To reduce the influence of order, the appearance of Xiao Li and Doctor Li was set to occur randomly. Subsequently, the participants completed a questionnaire on trust, acceptance, and mental workload. Finally, a short interview of the participants was conducted, comprising the following questions: (1) *Do you have any other questions for Xiao Li/Doctor Li?* (2) *Whom do you trust more, Xiao Li or Doctor Li? Why?* (3) *Do you have any suggestions or comments on the voice assistant?* The experimental procedure is shown in Fig. 3.

Mengyao Ma was the experimenter and interviewer (23-year-old female), and Runting Zhong trained the interviewer. As there was only one experimenter, we can ensure the consistency of the experiment. Prior to the experiment, the team discussed and categorised the responses provided by the older adults. During the experiment, the experimenter (Mengyao Ma) recorded the interview data using videos and notes. After the experiment, the interview data were first independently coded and classified into categories by the two research team members (Runting Zhong and Mengyao Ma). Then, the team members met and compared their coding differences. In the event of disagreement, the two team members discussed their reasoning and reached a consensus. Finally, the team counted the frequency and percentages of the participants’ opinions for each category using Microsoft Excel. The open-ended responses were analysed using magnitude coding, a process that quantifies the answers and highlights the most frequent comments [39, 40].

### Measures

All measurement items are provided in the Appendix Table 3. Trust was assessed using an 8-item trust scale with two subscales to address the trust of users in the ability and integrity of the voice assistant [14]. In addition, technology acceptance was assessed using a 5-point Likert scale (1 = totally disagree, 5 = totally agree) and included perceived ease of use, perceived usefulness, ease of learning, and intention to use [41–44]. Mental workload was assessed with the National Aeronautics and Space Administration – Task Load Index (NASA-TLX). The index has six major components (mental, physical and temporal demands, frustration, effort and performance) [45]. We used a 5-point Likert scale to measure each of the six components. An example question of the NASA-TLX questionnaire is as follows: “It takes brain power to complete the task”. Cronbach’s $\alpha$ for each dimension ranged from 0.547 to 0.879. For an exploratory study, a Cronbach’s $\alpha$ of over 0.6 is considered acceptable [46].

### Statistical analysis

Multivariate analysis of variance (MANOVA) was conducted to examine the effects of communication style and anthropomorphic setting. The within-subject variable was anthropomorphic setting (Xiao Li vs. Doctor Li), while the between-subject variable was communication style (social- vs. task-oriented). In addition, the covariates were five demographic variables (age, gender, living situation, use experience of voice assistant, and frequency of using digital products), which were included in the first MANOVA model. Among the demographic variables, frequency of using digital products significantly impacted the dependent variables. Therefore, we added frequency as an independent variable in a subsequent test. A *p value* < 0.05 was considered to indicate a significant difference. The effect sizes were calculated using the partial ${\eta }^{2}$. A partial ${\eta }^{2}$ of 0.01 was considered small, 0.09 medium, and 0.25 large [43, 47]. Statistical analysis was conducted using IBM SPSS version 26.0 (IBM, Armonk, NY, USA). Furthermore, the interview data regarding the recommendations by the participants were analysed using content analysis.

## Results

### Descriptive analysis

One hundred and six older Chinese adults (mean age = 71.8 years, SD = 4.6 years) participated in the study. The demographic information of the participants is presented in Table 2. There were no significant differences in demographic information (gender, age, living situation, use experience of voice assistant, frequency of using digital products) between the social-oriented and task-oriented groups (*p*s > 0.05). Fifteen participants (14.2%) could not read the script. Therefore, the researcher read the script for these participants, who repeated what they heard. None of the participants had previously used a smart speaker. However, 22 participants (20.8%) had used similar products, such as voice assistants for smartphones, automobiles, or televisions. In the subsequent analysis, we defined the weekly frequency of using digital devices as follows: “never use” was “low”; “1–7 times per week” was “medium”; “every day” was “high”.

**Table 2:** Demographic information of the participants (*N* = 106)

Variables	Total	Social	Task
**Gender**			
Male	48	26	22
Female	58	28	30
**Age (years)**	71.8 (4.6)	71.5 (4.3)	72 (4.9)
**Living situation**			
Lives alone	14	9	5
Lives with a partner	75	38	37
Lives with children	17	7	10
**Have you ever used voice assistant products?**			
Never use	84	42	42
Voice assistant for mobile phone	8	5	3
Voice assistant for automobile	1	1	0
Voice assistant for television	13	6	7
**Frequency of using digital devices**			
Never use	11	3	8
Use 1–7 times per week	87	40	37
Use every day	18	11	7

### Trust in ability

As presented in Table 3, a significant interaction effect between communication style and frequency was observed for trust in ability: *F* (2, 100) = 3.349, *p* = 0.039, ${\eta }^{2}$ = 0.063. Therefore, a simple main effect analysis is applied. For the social-oriented communication style, frequency had a significant effect on trust in ability: *F* (2, 100) = 4.638, *p* = 0.012, ${\eta }^{2}$ = 0.085. In addition, a Bonferroni post hoc test revealed that participants who used digital products daily had significantly higher trust in ability than those who used them 1–7 times per week (*p* = 0.019). Similarly, for the task-oriented communication style, frequency had a significant effect on trust in ability: *F* (2, 100) = 24.872, *p* < 0.001, ${\eta }^{2}$ = 0.332. Significant differences between frequency groups were observed (low vs. medium, *p* < 0.001; low vs. high, *p* < 0.001; medium vs. high, *p* = 0.011).

**Table 3:** Trust in ability

**Variables**	***M***	***SE***	***F***	***p***	${{\varvec{\eta}}}^{2}$
**Communication style**					
Social	4.20	0.067	18.285	< 0.001	0.155
Task	3.83	0.055			
**Anthropomorphic setting**					
Xiao Li	3.78	0.055	52.861	< 0.001	0.346
Doctor Li	4.25	0.054			
**Frequency**					
Low	3.63	0.102	18.380	< 0.001	0.269
Medium	4.04	0.034			
High	4.37	0.073			
**Communication style** $\times$ **Frequency**					
Social (Low)	4.00	0.174	3.349	0.039	0.063
Social (Medium)	4.16	0.048			
Social (High)	4.44	0.091			
Task (Low)	3.25	0.107			
Task (Medium)	3.93	0.050			
Task (High)	4.30	0.114			

### Trust in integrity

As presented in Table 4, a significant interaction effect between communication style and frequency was observed for trust in integrity: *F* (2, 100) = 12.49, *p* < 0.001, ${\eta }^{2}$ = 0.200. Therefore, a simple main effect analysis is applied. For the social-oriented communication style, frequency had no significant effect on trust in integrity: *F* (2, 100) = 0.656, *p* = 0.521, ${\eta }^{2}$ = 0.013. In contrast, for the task-oriented communication style, frequency had a significant effect on trust in integrity: *F* (2, 100) = 31.033, *p* < 0.001, ${\eta }^{2}$ = 0.383. Significant differences between the frequency groups of older adults were observed (low < medium < high; *p*s < 0.001), indicating that participants with a higher frequency of using digital devices had higher trust in the integrity of the health-related voice assistant.

**Table 4:** Trust in integrity

**Variables**	***M***	***SE***	***F***	***p***	${{\varvec{\eta}}}^{2}$
**Communication style**					
Social	4.26	0.066	18.31	< 0.001	0.155
Task	3.90	0.053			
**Anthropomorphic setting**					
Xiao Li	3.83	0.066	41.79	< 0.001	0.295
Doctor Li	4.32	0.046			
**Frequency**					
Low	3.80	0.099	9.553	< 0.001	0.160
Medium	4.10	0.033			
High	4.33	0.071			
**Communication style**$\times$**Frequency**					
Social (Low)	4.33	0.170	12.49	< 0.001	0.200
Social (Medium)	4.18	0.046			
Social (High)	4.26	0.089			
Task (Low)	3.27	0.104			
Task (Medium)	4.03	0.048			
Task (High)	4.39	0.111			

### Technology acceptance

As presented in Table 5, we observed a significant interaction effect between communication style and frequency for trust in technology acceptance: *F* (2, 100) = 5.842, *p* = 0.004, ${\eta }^{2}$ = 0.105. For the social-oriented communication style, frequency had a significant effect on acceptance: *F* (2, 100) = 5.240, *p* = 0.007, ${\eta }^{2}$ = 0.095. The post hoc analysis revealed that participants who used digital products daily had significantly higher trust in the ability of the voice assistant than those who used them 1–7 times per week (*p* = 0.005). In addition, for the task-oriented communication style, frequency had a significant effect on acceptance: *F* (2, 100) = 23.579, *p* < 0.001, ${\eta }^{2}$ = 0.320. Significant differences between the groups were observed (low vs. medium, *p* < 0.001; low vs. high, *p* < 0.001; medium vs. high, *p* = 0.026).

**Table 5:** Technology acceptance

**Variables**	***M***	***SE***	***F***	***p***	${{\varvec{\eta}}}^{2}$
**Communication style**					
Social	4.16	0.072	13.783	< 0.001	0.121
Task	3.82	0.058			
**Anthropomorphic setting**					
Xiao Li	3.81	0.051	70.37	< 0.001	0.413
Doctor Li	4.18	0.051			
**Frequency**					
Low	3.69	0.109	19.032	< 0.001	0.276
Medium	3.90	0.037			
High	4.38	0.078			
**Communication style**$\times$**Frequency**					
Social (Low)	4.13	0.186	5.842	0.004	0.105
Social (Medium)	4.01	0.051			
Social (High)	4.36	0.097			
Task (Low)	3.26	0.114			
Task (Medium)	3.80	0.053			
Task (High)	4.41	0.121			
**Anthropomorphic setting**$\times$**Frequency**					
Xiao Li (Low)	3.37	0.121	6.86	0.002	0.121
Xiao Li (Medium)	3.75	0.041			
Xiao Li (High)	4.30	0.087			
Doctor Li (Low)	4.02	0.120			
Doctor Li (Medium)	4.05	0.041			
Doctor Li (High)	4.47	0.086			

Furthermore, there was a significant interaction effect between anthropomorphic setting and frequency: *F* (2, 100) = 6.86, *p* = 0.002, ${\eta }^{2}$ = 0.121. For the anthropomorphic setting Xiao Li, frequency had a significant effect on acceptance: *F* (2, 100) = 23.277, *p* < 0.001, ${\eta }^{2}$ = 0.318. Post hoc analysis revealed significant differences between groups (low vs. medium, *p* = 0.011; low vs. high, *p* < 0.001; medium vs. high, *p* < 0.001). Moreover, for the anthropomorphic setting Doctor Li, frequency had a significant effect on acceptance: *F* (2, 100) = 23.579, *p* < 0.001, ${\eta }^{2}$ = 0.168. Participants who used digital products every day had significantly higher acceptance than those who never used digital products (*p* = 0.009) or used digital products 1–7 times per week (*p* < 0.001).

### Mental workload

As presented in Table 6, no significant interaction effects were observed between variables. The anthropomorphic setting had a significant effect on mental workload: *F*(1, 100) = 11.575, *p* = 0.001, ${\eta }^{2}$ = 0.104. Participants interacting with Xiao Li had a significantly higher mental workload than those interacting with Doctor Li.

**Table 6:** Mental workload

**Variables**	***M***	***SE***	***F***	***p***	${{\varvec{\eta}}}^{2}$
**Communication style**					
Social	1.61	0.118	0.003	0.960	< 0.001
Task	1.61	0.096			
**Anthropomorphic setting**					
Xiao Li	1.67	0.076	11.575	0.001	0.104
Doctor Li	1.55	0.080			
**Frequency**					
Low	1.66	0.179	0.751	0.474	0.015
Medium	1.67	0.060			
High	1.50	0.128			

### Interview analysis

#### Questions for Xiao Li/Doctor Li

Thirty-eight older adults participated in the interviews. Interviewees were randomly selected by the study team based on the participant's interests. Regarding questions for Xiao Li/Doctor Li, the participants mainly disclosed their physical conditions and diseases to obtain information on drugs, treatments, and nursing from the voice assistant. Participants mentioned that they wanted to ask Xiao Li/Doctor Li additional questions about their health conditions, such as hypertension. An example quote was, “*What medicine should I take for hypertension? What shall I do?*” The most frequently mentioned diseases were hypertension (7 participants), colds (5), gastrointestinal disease (4), eye disease (4), lumbar disease (3), leg trouble (2), postoperative care (2), insomnia (2), and COVID-19 (1). Regarding the illness-related information they provided, the participants mostly offered full details, and they even described certain illnesses in detail, including how they were contracted.

#### More trustworthy anthropomorphic setting

Eight participants mentioned that the voice assistant was very useful, and six considered Doctor Li more trustworthy than Xiao Li. One participant stated, “*Doctors must be professional, and I certainly trust them more*.” Another participant stated, “*I think Doctor Li is more professional than Xiao Li. After all, she graduated from a medical university and has experience*.” However, two participants believed that there was no difference between the two voice assistants because their answers were relatively fixed.

#### Suggestions for voice assistant developers

Four participants hoped that voice assistant developers would pay more attention to health care in the future: “*We are old now with pain all over the body, and I hope the developers of this [voice assistant] will pay more attention to health care*.” Three participants suggested that the voice assistant should be combined with real hospitals and doctors: “*It would be more convincing if a voice assistant can simulate face-to-face consultation with a doctor, tones, and words*.”

Participants provided suggestions regarding technological issues. Three participants desired their voice assistants to be more portable and able to be used without the internet. In addition, three participants noted the problem that the current range of use was not sufficiently wide: “*It would be great if the voice assistant could answer questions about the instructions for digital equipment, how to maintain electrical appliances, and how to operate digital equipment needed in travel*.” Considering that the memory of older adults declines rapidly, two participants suggested that the voice assistant could print the answers on paper to enable forgetful people to review the answers. Furthermore, one participant suggested a demonstration function: “*When encouraging an old man to exercise, a character could be simulated to exercise with the older adults to increase interest in the activity*.”

## Discussion

### General discussion

#### Social-oriented communication style is preferred in the health context

Regarding the effect of communication style, the participating older adults evaluated the social-oriented voice assistant significantly higher in trust in ability, integrity, and acceptance. Therefore, H1 was partly supported. The results of the current study differ from the previous studies [14, 48]. A possible reason is that the previous studies were conducted in the context of shopping, while this study was conducted in a health-related context. A social-oriented communication style may help older adults experience the impression of caring and empathy, thus gaining a more positive evaluation. In addition, a social-oriented communication style uses small talk, questions, feedback, and encouragement, which may result in the impression that the voice assistant is providing more information, such as showing a friendly attitude or offering a positive response to older adults.

There are differences between interacting with a voice assistant and interacting with real doctors. In the IoT environment, Chinese users wish to be dominant over things and to establish feelings of superiority and authority, which would indicate the user’s absolute control over things. The user is the “boss”, and the things are “obedient and smart subordinates” that provide the user support [49]. However, in real patient-doctor communication, the responses of patients to decision-making and information sharing are distinctively doctor-centred [50]; i.e., the doctor is the dominant individual in the communicative exchange. This phenomenon has an important implication for designing elderly friendly IoT health-related services. That is, when designing an IoT health-related service for older Chinese, the conversation design should differ from that of real patient-doctor communication, and a social-oriented communication style should therefore be preferentially considered.

#### Voice assistants with medical backgrounds are preferred in a health context

Regarding the effect of anthropomorphic setting, the analysed older adults evaluated the voice assistant with a medical background (i.e., Doctor Li) more positively. That is, they had higher trust in Doctor Li’s ability and integrity as well as higher technology acceptance and a lower mental workload with Doctor Li, although the communication content was consistent with that used for the other assistant. Therefore, H2 was supported. According to [51], people more easily accept robots that are in line with their stereotypes regarding such devices. This finding indicates that a stereotype of real-life experiences may also be generalised to an anthropomorphic setting in a smart environment. Older adults would trust an “expert” more in the smart environment in terms of communicating regarding health-related issues. The anthropomorphic setting of the “doctor” caused the participating older adults to feel that this voice assistant was more professional. These results confirm that the use of stereotypes that correspond to a specific context is an effective way to improve the trust of older adults in voice assistants.

This study found that the participating older adults had a higher level of trust in health-related voice assistants. The participants were willing to disclose detailed personal information, such as the type of disease they were suffering from. However, considered from another perspective, overtrust may also raise privacy concerns. Overtrust means that individuals blindly follow the voice assistant or take risks because they believe the voice assistant can perform functions that it does not have [52]. Therefore, it is necessary to include privacy protection mechanisms in the design of products and services for older adults, such as reminders regarding them how to answer, appropriate behaviour, and avoiding risky behaviour.

#### Older adults with a higher frequency of using digital products evaluated voice assistants more positively

This study also found that older adults with a higher frequency of using digital products evaluated the voice assistants more positively. That is, they had higher trust in the ability and integrity of voice assistants and exhibited higher technology acceptance. Age, gender and living situation did not significantly influence user evaluation. Therefore, H3 was partly supported. This result is in line with Ghorayeb et al. [30]. For most older adults, a voice assistant is a new IoT technology. Therefore, a lack of prior experience in using digital products may cause older adults to be doubtful about the performance of voice assistants. Thus, for older adults, previous experience using digital devices is beneficial for the adoption of voice assistants in the first interaction. To bridge the digital divide between older adults and youth, it is necessary for older adults to take the first step in using a voice assistant. In practice, sharing experiences with peers and children could serve as an important way to enhance the understanding of and trust in voice assistants of older adults.

### Implications

This study investigated the attitudes of Chinese older adults towards voice assistants in a health-related context. Specifically, we investigated the effect of communication style and anthropomorphic setting on the trust, acceptance, and mental workload of older adults. The study participants were older Chinese adults who were using voice assistants (smart speakers) for the first time. We found that the participants had a relatively high level of trust in and acceptance of the voice assistant in a health-related context. In addition, the participants held more positive attitudes towards a social-oriented communication style and a voice assistant with a medical background. Furthermore, those participants with a higher frequency of digital product use had a more positive attitude towards the voice assistant. Therefore, when designing a voice assistant for older adults in the health context, it is necessary to create an assistant that requires older adults to interact with it. Adopting a more social-oriented communication style (for example, providing guidance, using small talk, asking questions, and furnishing feedback and encouragement) and providing an anthropomorphic setting for the voice assistant in which the assistant has a medical background (for example, that of a doctor, nurse, or fitness coach) could help older adults trust and accept the voice assistant more. Furthermore, functions such as connecting with real hospitals and doctors, portability, offline accessibility, operation guidance, printing, and a demonstration function are also requirements for older adults. The results of this study reveal the preferences of older adults for voice assistants in an intelligent environment and provide suggestions for improving the user experience of older adults utilizing voice assistants.

### Limitations and future studies

Our study has several limitations. First, it only included community-dwelling older adults from a city in China, and the dialogue script was localised according to traditional medical beliefs in terms of diet, daily practices, and medication use [53–55]. Therefore, generalisation of the results requires caution. Future studies can examine whether older adults prefer a voice assistant that acts like a traditional medicine doctor rather than a modern medical doctor.

Second, the study used the “Wizard of Oz” method to simulate communication between older adults. The responses of the participating older adults may differ from those in a realistic setting. Future studies should validate the conclusions of this study in an actual smart environment.

Third, we investigated the effect of communication style and anthropomorphic setting in a health care context. Future studies could examine whether the effect of communication style and anthropomorphic setting could be generalised to other use contexts, such as educational or daily life contexts.

## Conclusions

This study investigated the effect of the communication style (socially-oriented vs. task-oriented) and anthropomorphic setting (ordinary profession vs. medical background) of a voice assistant on the trust (ability and integrity), acceptance, and mental workload of older adults using such an assistant. The results indicated that older adults preferred a social-oriented voice assistant and a voice assistant with a medical background. In addition, older adults with more digital product experience evaluated voice assistance more positively. Furthermore, the analysed older adults identified functions such as connecting with real hospitals and doctors, portability, offline accessibility, operation guidance, printing, and demonstration functions as requirements. The results suggest that when designing a voice assistant for older adults, using a social-oriented communication style and providing an anthropomorphic setting in which the voice assistant has a medical background are effective ways to improve the trust and acceptance of voice assistants. Our results have implications for designers seeking to improve the acceptability and trust of older adults and support ageing in place. 

## Supplementary Information

**Additional file 1: Table A1.** Design of the voice assistant interaction (Task Script: Exercise)** Table A2.** Design of the voice assistant interaction (Task Script: Medical consultation)** Table A3.** Questionnaires used in this study

## Data Availability

The materials and data to support the study findings are available from the first author [RZ] upon request.

## Abbreviations

IoT: Internet of things
MANOVA: Multivariate analysis of variance
NASA-TLX: National Aeronautics and Space Administration-Task Load Index
PDF: Portable Document Format
GDP: Gross domestic product
M: Mean
SD: Standard deviation
SE: Standard error
CI: Confidence interval
VA: Voice assistant
P: Participant
