# Supplementary material for: Effects of communication style, anthropomorphic setting and individual differences on older adults using voice assistants in a health context
Source: BMC Geriatr. 2022 Sep 15;22:751. doi: 10.1186/s12877-022-03428-2 (PMC9473738; doi:10.1186/s12877-022-03428-2)
Supplement: Supplementary file 1 — Additional file 1: Table A1. Design of the voice assistant interaction (Task Script: Exercise) Table A2. Design of the voice assistant interaction (Task Script: Medical consultation) Table A3. Questionnaires used in this study [file 12877_2022_3428_MOESM1_ESM.docx]

# Appendix

**Table A1** Design of the voice assistant interaction (Task Script: Exercise)

| Task-oriented | Social-oriented |
| --- | --- |
| **VA:** Hello, this is Xiao Li (Doctor Li), can I help you?  **P:** What are the benefits of chest expansion?  **VA:** Chest expansion exercise has the following benefits: First of all, it can correct abnormal curvature of cervical spine, prevention and treatment of cervical spondylosis. Secondly, chest enlargement exercise can dredge heart, lung and blood vessels, promote blood circulation, improve heart and lung function.  **P:** What should I pay attention to when doing chest enlargement exercise?  **VA:** Breast enlargement exercise has the following needs to pay attention to: first, women and the elderly, it is recommended not to use external equipment. Second, jogging, push-ups collocation chest expansion exercise, can improve the lung capacity of the person, the effect of cardiopulmonary function is better. Third, chest enlargement may cause abdominal pain in patients with lung diseases.  **P:** Please set an alarm clock for three o 'clock.  **VA:** Okay, the 3:00 alarm is set.  **P:** Teach me how to do breast expansion.  **VA:** Please cross your hands and make a fist, parallel to your shoulders, with both arms firmly open to the outside of your body, hold for 2 seconds, then return to the crossed position. Do it 5 times in a set. | **VA:** Hi! This is Xiao Li (Doctor Li), I am so happy to see you today! Do you have any questions for me? I look forward to helping you solve them!  **P:** What are the benefits of chest expansion?  **VA:** Chest expansion movement has a lot of benefits to the body, I say one by one to you: first of all, it can correct abnormal curvature of cervical spine, prevention and treatment of cervical spondylosis. Secondly, chest enlargement exercise can dredge heart, lung and blood vessels, promote blood circulation, improve heart and lung function. How about that? Doesn’t that sound great? Let’s exercise together!  **P:** What should I pay attention to when doing chest enlargement exercise?  **VA:** Breast enlargement exercise has the following needs to pay attention to, must be careful ah: first, women and the elderly, it is recommended not to use external equipment. Second, jogging, push-ups collocation chest expansion exercise, can improve the lung capacity of the person, the effect of cardiopulmonary function is better. Third, chest enlargement may cause abdominal pain in patients with lung diseases. How about, after listening to is there a lot of inspiration? Go and try it!  **P:** Please set an alarm clock for three o 'clock.  **VA:** All right, the alarm is set for 3:00. I will certainly remind you on time!  **P:** Teach me how to do breast expansion.  **VA:** Next, let's complete the chest expansion exercise together! Make a fist with your hands crossed, parallel to your shoulders, and stretch your arms out to the outside of your body. Hold for 2 seconds, then return to the crossed position. Do it 5 times in a set. |

**Table A2** Design of the voice assistant interaction (Task Script: Medical consultation)

| Task-oriented | Social-oriented |
| --- | --- |
| **VA:** Hello, this is Xiao Li (Doctor Li), can I help you?  **P:** What are the symptoms of a cold?  **VA:** The symptoms of wind-chill cold are severe chills, mild fever, no sweat, headache, sore limbs, even pain, heavy nasal congestion, sneezing, runny nose, itching pharynx, cough, thin sputum; Tongue coating thin white, pulse floating or floating tight.  **P:** What does the medicaments that treats chill cold have?  **VA:** The treatment of wind-cold is based on proprietary Chinese medicine, because wind-cold is a syndrome of Traditional Chinese medicine, clinical treatment of wind-cold proprietary Chinese medicine is relatively rare, the common is GanMaoLingKeLi (999). | **VA:** Hi! This is Xiao Li (Doctor Li), I am so happy to see you today! Do you have any questions for me? I look forward to helping you solve them!  **P:** What are the symptoms of a cold?  **VA:** Are you sick? Don't worry, I will tell you what the symptoms of cold, severe chills, fever is light, no sweat, headache, sore limbs, even pain, heavy nasal congestion, sneezing, runny nose, itching pharynx, cough, thin sputum; Tongue coating thin white, pulse floating or floating tight. Check whether you are sick, if so, be sure to see a doctor soon!  **P:** What does the medicaments that treats chill cold have?  **VA:** The treatment of wind-cold is based on proprietary Chinese medicine, because wind-cold is a syndrome of Traditional Chinese medicine, clinical treatment of wind-cold proprietary Chinese medicine is relatively rare, the common is GanMaoLingKeLi (999). I hope this information will be helpful to you. If it is serious, you must see a doctor soon. |

**Table A3** Questionnaires used in this study

| Measures | Factors (if multi-dimensional) and Items. | Sources | Cronbach’s $\alpha$  (Xiao Li) | Cronbach’s $\alpha$  (Doctor Li) |
| --- | --- | --- | --- | --- |
| **Trust** | **Ability** |  | .695 | .728 |
|  | This voice assistant competently and effectively interacted with me. | [14] |  |  |
|  | This voice assistant performed all of its roles very well. | [14] |  |  |
|  | Overall, this voice assistant was capable and proficient. | [14] |  |  |
|  | In general, this voice assistant was informative. | [14] |  |  |
|  | **Integrity** |  | .748 | .603 |
|  | This voice assistant was truthful in its dealing with me. | [14] |  |  |
|  | I would characterize this voice assistant as being honest. | [14] |  |  |
|  | This voice assistant kept its commitments. | [14] |  |  |
|  | This voice assistant was sincere and genuine. | [14] |  |  |
| **Technology Acceptanc**e | **Perceived ease of use** |  | .703 | .631 |
|  | I think it is easy to use a voice assistant. | [41, 43] |  |  |
|  | I think I know how to control voice assistant to talk and give voice commands. | [44] |  |  |
|  | **Perceived usefulness** |  | .636 | .641 |
|  | Voice assistants are very useful. | [41, 43] |  |  |
|  | It is enjoyable to use a voice assistant. | [44] |  |  |
|  | Voice assistant can make my life more convenient, comfortable, and efficient. | [44] |  |  |
|  | **Ease of learning** |  | .600 | .547 |
|  | Learning to use a voice assistant is easy. | [42] |  |  |
|  | I want to learn how to use a voice assistant. | [42] |  |  |
|  | **Intention to use** |  | .674 | .634 |
|  | I am interested in using a voice assistant. | [41, 43] |  |  |
|  | If I have a voice assistant, I tend to use a smart product associated with it. | [41, 43] |  |  |
|  | If I had a voice assistant, I would frequently use it. | [44] |  |  |
| **Mental Workload** | It takes brain power to complete the task. | [45] | .833 | .879 |
|  | It takes physical energy to complete the task. | [45] |  |  |
|  | There is a lot of time pressure to complete the task. | [45] |  |  |
|  | It feels pointless to complete the task. | [45] |  |  |
|  | It takes a lot of effort to complete the task. | [45] |  |  |
|  | It’s frustrating to complete the task. | [45] |  |  |

Note: Answers 1 (Totally disagree) to 5 (Totally agree)
